# Impact of COVID-19 Pandemic on Pathology Residents/Trainees in North America: A Survey-Based Study

**DOI:** 10.7759/cureus.40967

**Published:** 2023-06-26

**Authors:** Satyapal Chahar, Lomesh Choudhary, Ram Ahuja, Anita Choudhary

**Affiliations:** 1 Pathology/Gastrointestinal and Liver Pathology, University of Mississippi Medical Center, Jackson, USA; 2 Biomedical Sciences, York University, Toronto, CAN; 3 Internal Medicine, Flowers hospital, Dothan, USA

**Keywords:** covid 19, pathology, pathology practice, pathology teaching, medical resident education

## Abstract

Background

The COVID-19 pandemic has had a significant impact on resident training and education in the field of Pathology. This study aims to identify the tangible effects and resultant changes in education for Pathology trainees that have resulted from the pandemic.

Design

An electronic survey regarding Pathology trainee perceptions and experiences in relation to COVID-19 was created via Google Forms. The questionnaire was distributed to the pathology trainees via Twitter and email. The survey was also shared with all Pathology residency program coordinators across the USA and Canada.

Results

One hundred forty-five trainees responded to the questionnaire. 37.6% reported a significant decrease in specimen volume, whereas 43.3% reported a slight decrease in specimen volume. 18.3% reported the cancellation of educational lectures before shifting to a virtual platform for didactic purposes. However, 74.6% reported shifting all educational activities to virtual platforms. 35% cited cancellations of grand rounds, whereas 18.2% reported cancellations of grand rounds led by guest speakers. 53.5% took COVID-19 tests, and 22.7% were quarantined. 100% reported a change in sign-out culture.

Conclusions

This pandemic has significantly impacted pathology training in various aspects, including training, education, and well-being. Residents harbored anxiety and stress regarding board exam delays or uncertainties, inadequate exam preparation time, family separation, and compromised safety. The exact quantification of educational loss varied from program to program. A significant decrease in specimen volume and detrimental changes in sign-out culture are indicators of compromised resident education due to the pandemic. This pandemic has extended the use of digital pathology and virtual platforms to a higher extent. Free virtual educational resources provided by various pathology organizations were critically important interventions during this pandemic, contributing to resident education. The pandemic has shown that developing a comprehensive infrastructure to overcome the loss of educational opportunities is of paramount importance to alleviate stress and anxiety among trainees.

## Introduction

The COVID-19 pandemic has possibly affected almost every human life on the planet in several ways, either directly or indirectly. Similarly, pathology training programs have not been spared by the pandemic. COVID-19 has resulted in the disruption of medical education, leading to technological innovation and the utilization of online learning platforms [1-3]. The decrease in surgical specimens and the restructuring of traditional curricula to online formats have greatly affected pathology trainees [4,5]. The pandemic has not only impacted education but has also disrupted the well-being of the trainees and their immediate future plans.

On January 20, 2020, the Centers for Disease Control and Prevention (CDC) confirmed the first laboratory-confirmed COVID-19 case in the United States [5]. The healthcare system faced the catastrophic effects of the COVID-19 pandemic, resulting in a healthcare crisis worldwide [6-10]. The enormous influx of hospitalizations due to the coronavirus disease overwhelmed hospital systems, leading to shortages of personal protective equipment (PPE), ICU beds, limited availability of ventilators, and staff shortages [11-13]. A triage approach disrupted outpatient care, leading to increased cancellations of surgeries, long-term care, home care, and cancer care. These measures aimed to channel medical supplies, including PPE and workforce, toward critically ill patients requiring hospitalization [14]. Similarly, in Canada, the universal healthcare system faced major challenges. Paramount efforts and emphasis were made to slow down the spread in order to reduce the burden on the already overwhelmed healthcare system. The U.S. Center for Disease Control and Prevention issued guidelines for social distancing, isolation, and quarantine in an effort to flatten the curve [15,16].

This unprecedented circumstance affected every aspect of conventional medical practice training, including pathology [17]. Even though pathology trainees were not directly involved in providing care to COVID-19 patients, they were uniquely impacted by the pandemic. The cancellation of elective and non-emergency surgeries led to a decrease in surgical pathology specimens [18]. On the other hand, efforts were made to develop and provide COVID-19 testing, resulting in a large influx of specimens in clinical pathology [19]. Hospitals across the country changed residents' schedules to provide a workforce where it was most needed while reducing unnecessary exposure and the risk of spreading infection among house staff, as well as judiciously using healthcare resources like PPE [19].

Our team conducted an anonymous survey of pathology trainees in the United States and Canada to better understand the impact and extent of the pandemic's effect on pathology training. The survey explored the learning strategies implemented by training programs to recover from the losses caused by the pandemic. The study focused on examining the impact on education, well-being, and future plans, and discussed potential measures to mitigate the immediate and long-term effects of the pandemic. The emergence of new variants of the SARS-CoV-2 virus has extended the period of uncertainty and delayed the return to normalcy. The results are expected to provide meaningful insights not only to the trainees but also to program leadership and faculty, helping them understand the extent of the impact on pathology training. They can then identify the most vulnerable areas and tailor an approach to ensure a successful recovery from the impact of the pandemic.

## Materials and methods

An anonymous survey was created using Google Forms to collect data for our study. The form provided a brief description to the participants, explaining the purpose of the study and the relevance of the survey questions. The survey consisted of twenty-nine questions, including multiple-choice, short-answer, and long-answer formats (Appendices). To participate, individuals had to meet the inclusion criteria of being current pathology trainees (residents and fellows) in pathology training programs in the United States or Canada.

The questionnaire collected demographic information from participants, including the location of their training program, training type (anatomical pathology/AP, clinical pathology/CP, Anatomic and clinical pathology/APCP), size of the training program, type of training program (University program, University-affiliated community program, or community program), and training status (resident or fellow). Additionally, the survey included questions about the impact of the COVID-19 pandemic on pathology training, such as changes in duty schedules, educational activities, sign-out practices, specimen volume, non-pathology-related duty assignments, fellowship applications, and personal well-being. Participants were also encouraged to share any unique or innovative experiences they had during this time.

Once the initial survey was developed, all authors reviewed and made edits to create the final survey questionnaire. Efforts were made to ensure the survey focused specifically on pathology training and the effects of the COVID-19 pandemic. The anonymous survey link was then sent to all pathology residency program coordinators in the United States and Canadian pathology training programs. These coordinators distributed the survey among their pathology trainees, including both residents and fellows. A reminder email was sent two weeks later to encourage participation.

## Results

A total of 145 pathology trainees participated in the survey. To analyze the distribution of survey responses, information regarding the geographical location of the participants (Table 1) and the size of their training programs (Table 2) was collected. The data revealed that a significant portion of respondents (29.5%) were from the east coast, and approximately 10% of participants were Canadian (Table 1). In terms of program size, the largest group of respondents (approximately 30%) hailed from programs with 15-20 residents, while only 12.6% came from training programs with less than 10 residents (Table 2).

**Table 1:** Geographical distribution of respondents

Geographical distribution	
East coast	29.5%
Mid-west	29.5%
South	26%
Canada	10%
West Coast	5%

**Table 2:** Size of training program of respondent

Size	
>25	15.4%
20-25	25.2%
15-20	30%
10-15	16.8%
<10	12.6%

Among the respondents, the majority (47.2%) were APCP residents, followed by AP-only residents comprising 12.5% of the total participants. Trainees in the transition from residency to fellowship training accounted for 18.8% of the respondents, while 11.1% had transitioned from one fellowship to another (Table 3).

**Table 3:** Respondent’s training status during this period

Training status	
Anatomic and clinical pathology	47.1%
Transition from residency to fellowship	18.8%
Anatomic pathology only	12.5%
Transition from fellowship to fellowship	11.1%
Transition from training to job	4.9%
Started residency on July 1, 2020	3.5%
Transition from fellowship to residency	0.7%
Transition from job to fellowship	0.7%
Clinical pathology only	0.7%

Educational activity

Regarding educational activities where pathology trainees were responsible for presenting at academic conferences, 59.2% of the respondents stated that their presentations were shifted to virtual platforms instead of being held in person. In contrast, 7.7% reported that their presentations were canceled. On the other hand, two participants noted an increase in presentations given by residents.

**Figure 1:**
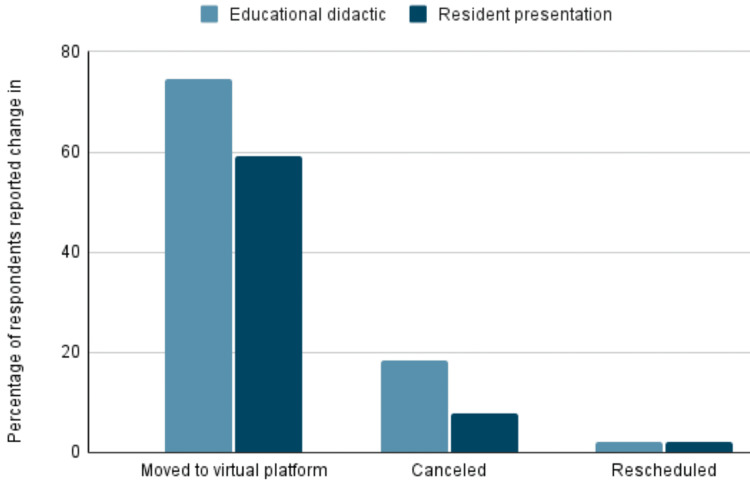
Resident presentation and educational didactic

In terms of educational lectures, the majority of respondents (74.6%) reported that all lectures were transitioned to virtual platforms. Among the participants, 18.3% witnessed the cancellation of a few educational lectures before moving the activities to a virtual platform, while 2.1% reported the cancellation of all lectures while remaining 5% did not comment about it (Figure 1).

Additionally, changes were observed in departmental educational grand rounds. Around 35% of respondents were informed about the cancellation of all grand rounds, while 18.2% reported the cancellation of grand rounds only if the guest faculty was traveling from another city.

Surgical pathology workload and sign-out practice

**Figure 2:**
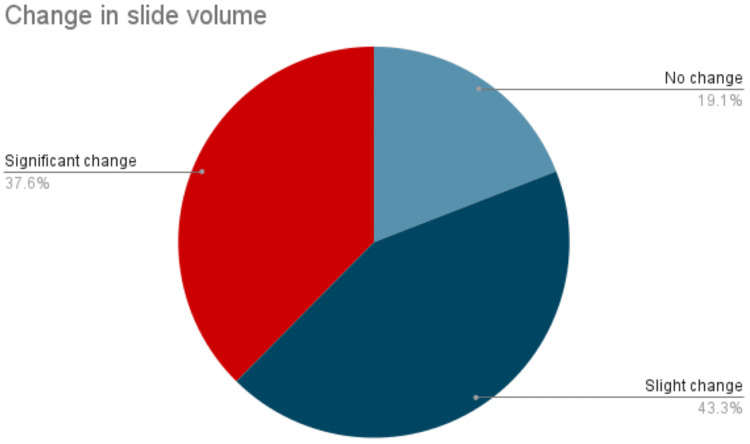
Degree of change in the surgical pathology slide volume during pandemic

The cancellation of non-emergent and elective surgeries had a significant impact on the volume of surgical pathology cases, leading to a reduction in workload. 80.9% of the respondents observed a decrease in surgical pathology cases during the pandemic. Among this 81%, 37.6% reported a substantial reduction in cases, while 43.3% reported a slight decrease (Figure 2).

**Figure 3:**
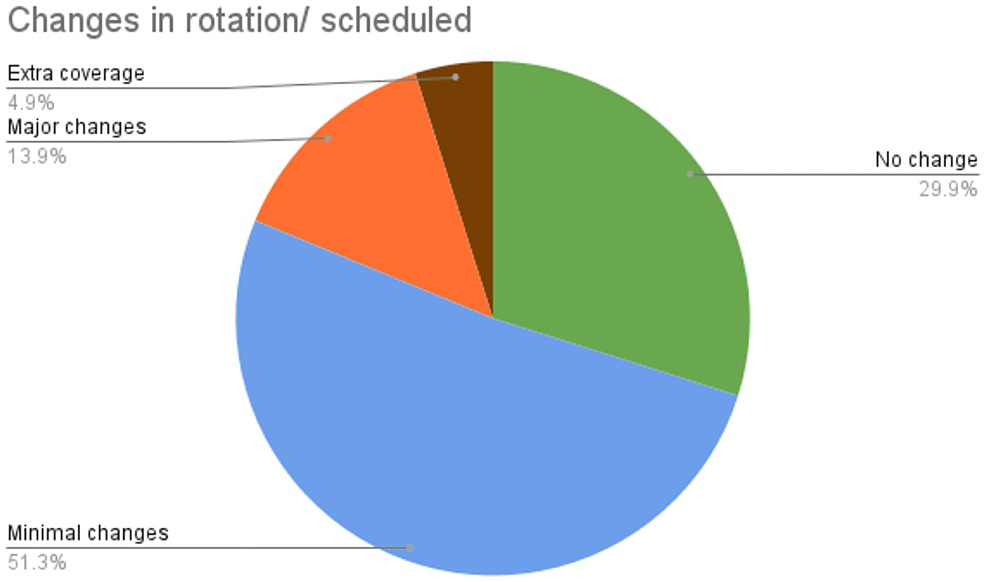
Changes in rotation/scheduled

The pandemic also necessitated changes in sign-out practices to ensure social distancing. Respondents reported various sign-out methods, including virtual-only sign-out (22.5%), passing slides to the attending physician for preview and receiving feedback via phone or email (11.3%), sign-out at multi-headed scopes to maintain social distancing (12%), and sign-out at dual-headed scopes with masks or see-through partitions (16.2%). Additionally, 33.8% of participants experienced multiple combinations of sign-out modalities depending on the attending physician and service. The pandemic also forced necessary changes in the residents' schedules to minimize exposure and redistribute the workload (Figure 3).

To combat for lost educational opportunity

Trends of effort were observed among the survey respondents as they sought to compensate for the lost educational opportunities. A significant portion, 33.6% of participants, utilized available online virtual slides to aid their studies. Additionally, 18.9% turned to study set slides. However, a notable proportion (16.8%) expressed their belief that they were unable to fully make up for the educational opportunities they had lost.

Many residents turned to social media platforms in search of any available educational resources. Among the participants, 34.7% reported following pathology-related content on social media, although they rarely posted themselves. Conversely, 25% of the participants stated that their social media activity remained unchanged during the pandemic.

The pandemic compelled trainees to rely on online resources during periods of downtime in the surgical pathology service. In the survey, 35.7% of respondents reported an increase in their use of online resources during the pandemic, while 7.7% reported a significant surge in their reliance on such resources. Notably, 47.6% of participants mentioned frequently utilizing online resources in their day-to-day practice, irrespective of the pandemic.

Effect on fellowship application, boards, and future plans

In the survey, 24 participants reported a significant impact of the pandemic on their plans to take the board certification exam administered by the American Board of Pathology (ABP). Half of the candidates who were planning to take the exam withdrew due to safety concerns and uncertainty surrounding the exam dates. The remaining 50% experienced delayed exams, which were eventually conducted virtually. Some participants expressed anxiety about the sudden change in the exam format and their unfamiliarity with virtual exam procedures.

Furthermore, fellowship applications and job applications were also affected by the unique circumstances imposed by COVID-19. The study included 44 participants who went through the application process during this period. The majority of respondents (40 out of 44) reported that their interviews were converted to virtual or telephone format instead of in-person. Approximately 20% of trainees mentioned that the pandemic had some impact on their future plans. Ten respondents reported a significant change in their fellowship choices as they were compelled to pursue alternative fellowships due to hiring freezes implemented by multiple facilities during the pandemic. Other reported challenges included the cancellation of away electives, delays in board exams, and the cancellation of the USMLE step 3.

Personal and family wellbeing

During the unprecedented and challenging times of the pandemic, pathology trainees were understandably concerned about the well-being of themselves and their families. Among the participants surveyed, 53.5% (76 individuals) underwent COVID-19 testing during this period. Out of these, 25 participants were tested due to mandatory hospital policies, while six participants were part of a study group requiring testing. The remaining 45 participants chose to take the test out of self-concern and due to the availability of free testing offered by their workplaces.

Additionally, 22.7% of the survey respondents had to undergo quarantine at some point during the pandemic. Furthermore, 35 pathology trainees in this survey faced the daunting challenge of taking care of their children amidst the closure of daycares and schools. The trainees were deeply concerned about transmitting the infection to their family members, which resulted in temporary family separations. Alarmingly, 43.8% of the respondents reported some form of family separation in order to prevent the spread of the infection to their loved ones.

Pregnant pathology trainees faced unique challenges during this period. The study identified eight participants who reported being pregnant during the pandemic. Among them, two participants were allowed to work from home by their program, two took time off to ensure their safety, one participant experienced no changes in rotations to minimize the risk of exposure, and two participants noted that no special arrangements were made for them during this time. Overall, the pandemic posed significant challenges and concerns for pathology trainees, impacting their personal lives, family dynamics, and work arrangements.

The overall effect on residency

During the pandemic, programs were compelled to implement changes in the schedules of pathology trainees in order to redistribute the workforce and prevent the spread of the virus. According to the survey conducted, approximately 70% of participants encountered modifications in their training schedules amidst the pandemic. Furthermore, pathology trainees who were transitioning from residency to fellowship or those who had recently commenced their residency encountered several challenges. Among the respondents, six individuals were unable to commence their fellowship on schedule, and two respondents faced delays in commencing their residency due to pandemic-related obstacles such as mandatory quarantines, travel bans, or visa processing delays.

## Discussion

Our team conducted an anonymous survey among pathology trainees across North America to assess the impact of the COVID-19 pandemic on pathology training, education, and well-being. This survey provided us an opportunity to gather comprehensive data from pathology trainees who have first-hand experienced the pandemic impact on their training and well-being.

We observed educational conferences presented by trainees were canceled before they were transitioned to virtual platforms. A similar trend was noted in the educational lectures. In addition, grand rounds by visiting faculty were canceled due to travel restrictions and institutional policies. As a result, trainee education was halted for a brief period. It was difficult to estimate the duration of this period as it varied from program to program before they established a smooth transition to the virtual platform. While these virtual meetings and platforms are fairly common in the corporate industry, it was a new and steeper learning curve for the medical field. Establishing a secure and protected technology to protect patient identity has prolonged the transition to the virtual platform.

A decrease in surgical pathology specimens due to cancellations of elective and non-emergent surgeries had resulted in a significant impact on learning [5]. In order to mitigate the spread of COVID-19, the traditional dual-headed scope sign-out was either removed, or its duration was significantly minimized. Alternative sign-out modalities were utilized, including virtual sign-outs, feedback via phone or email, or a combination [20]. Despite these alternative sign-out modalities, an overall decrease in sign-out time alongside a reduced number of specimens had an enormous effect on pathology training. However, these challenges provided an excellent opportunity to revolutionize the use of digital pathology and learning tools [21-23].

Efforts were made by trainees to recover from the educational loss resulting due to pandemic. Most of them utilized virtual slides to enhance their diagnostic skills. However, a significant portion of participants believed that the loss of education is so grave that it’s difficult to recover. The pandemic had accelerated the use of social media for pathology education [24-26]. Our study also observed a sharp increase in the usage of social media by trainees.

A timely intervention by a major pathology organization by providing extensive virtual lecture series, conferences, board review courses, and study materials greatly benefitted the trainees. We observed trainees made excellent use of free educational lectures, conferences, and study materials provided by major pathology organizations.

Traditionally, pathology programs do not participate in the match for fellowship recruitment. A standardized application for pathology fellowships provided by the College of American Pathologists has been adopted by many programs. However, many programs still have their own application process and deadline which imposes a unique challenge to the applicant resulting in a more painful and laborious application process. Our study observed that 45 participants had their interviews scheduled during this period of the COVID-19 pandemic. The majority of these interviews were conducted virtually or via phone interviews and few reported cancellations of interviews. The unfamiliarity of virtual interview platforms at the beginning of the COVID-19 era and the inability to visit the program in person has forced some candidates to stay in the same geographical location rather than rely on a short virtual interview to select their future fellowship training program. The uncertainty caused by COVID-19 affected not only the candidates but also the programs. As a result, programs had early fellowship application deadlines and offered positions sooner compared to the previous year, which shattered many dreams to get fellowship training at a program of choice.

Financial turmoil imposed by the pandemic led to a hiring freeze at many healthcare institutions across the country [27]. Ten participants were forced to pursue another fellowship due to unsuccessful attempts to secure a job amid a hiring freeze.

In the past, every pathology board taker needed to travel in person to the ABP center in Tampa, Florida, or Tuscon, Arizona. Travel restrictions and safety concerns during the pandemic made it impossible to conduct board exams traditionally without compromising safety. Fifty percent of board-going candidates from our study withdrew from the exam due to safety concerns and uncertainty regarding the exam date. The ABP conducted the exam online at remote locations to ensure the safety and well-being of its diplomat [28]. The Canadian Anatomical Pathology certification examination saw the removal of oral and practical components due to the pandemic. The majority of residents agreed with the changes to the examination [28]. In our survey, the unfamiliarity with this new exam format resulted in significant distress among the board takers. Securing a silent space for a three-day virtual remote exam was challenging for some candidates, especially for those with young children, pets, and partners working from home during the pandemic. A decrease in AP board exam pass rates was seen, with an 85% pass rate in 2020 to a decreased 82% in 2021. However, CP board exam pass rates climbed from 88% in 2020 to 94% in 2021. A significant decrease in medical autopsy was observed during the pandemic, which not only compromised pathology education but also imposed a challenge to achieve the autopsy numbers required for board eligibility. However, the ABP acknowledged this challenge in a timely fashion and rescued the board takers by lowering the required number of autopsies to 30 for board eligibility [29].

We have observed in our study the biggest challenge pathology trainees faced during the pandemic, which affected their personal well-being, was family separation, especially for those whose families resided in different geographical locations. Childcare imposed the paramount challenge amid daycare and school closure and must seek help from friends and family. Pregnant pathology trainees faced a unique challenge due to unclear data regarding the vertical transmission of infection.

The limitation of our study is the low response rate. The survey was emailed to all pathology program coordinators in the US and Canada. It was at the coordinators discretion if the survey was shared with pathology trainees or not. We have no way to determine the number of coordinators who have shared the survey with trainees. In context, there are 2,274 active pathology residents in the United States in 2021; however, only 5.7% (130 US residents) responded to the survey. Even though it was a low response rate, participants of the survey were widely distributed across North America and were at different levels of their training from PGY-1 to fellowship. The wide geographical distribution and various levels of training provided us with a broad perspective of the challenges faced by trainees during a pandemic.

Despite many deleterious effects of the pandemic, it has helped us to surmount the inertia to use unfamiliar technology and possibly caused a permanent change in the landscape of pathology practice. It has certainly accelerated the induction of digital components in our day-to-day practice, the use of social media for educational activity and professional networking, and the growing use of virtual interviews, academic conferences, and remote and computer-based board exams. The COVID-19 pandemic has provided momentum to reshape traditional pathology practice and training for its future advancement.

## Conclusions

The pandemic significantly impacted pathology training, causing anxiety and stress among trainees due to delays in board exams, limited preparation time, family separation, and compromised safety. Surgical pathology education faced challenges with a decrease in specimen volume and negative changes in sign-out practices. However, the pandemic also accelerated the adoption of digital pathology and virtual platforms, with free virtual resources playing a crucial role in supporting pathology education. Developing a comprehensive infrastructure to address educational loss is necessary to alleviate trainees' stress, while a comprehensive analysis of the pandemic's long-term effects will enable tailored interventions to mitigate its impact.

## Appendices

**Table 4:** Questionnaire for pathology trainees - The Impact of the COVID-19 Pandemic on Pathology Training

Question Number	Question for pathology Trainees
1.	What type of training have you been enrolled in during this time period? o Started residency from July 1, 2020 o APCP resident o AP only resident o CP only resident o Transition from residency to fellowship o Transition from fellowship to fellowship o Transition from pathology training to job o Other…
2.	Location (state/province/city) of your training program? (If you have made a transition, please mention the location of both the programs.)
3.	What is the size of your training program? o <5 o 5-10 o 10-15 o 15-20 o 20-25 o >25
4.	Have you been quarantined during this period? o Yes o No
5.	Have you been pulled to perform clinical duties beyond the scope of pathology training, due to an increased patient load during a COVID-19 peak in your area? o No o Yes o Kept as a backup o Just a preparedness alert that they might need pathology trainees' help o I volunteered o Other…
6.	Please specify any duties assigned to you beyond pathology training. o Not applicable o Medicine floor o ICU o Sample collection o Emergency room o Other… o How did you prepare yourself for assigned clinical tasks? o Not applicable o Hospital provided a workshop o Team up with resident of a clinical specialty o I had an internship training in a clinical specialty o I had a residency training in a clinical specialty o Other…
7.	How did this affect the duration of your training? o No effect o It extended my training o I wasn't able to start my residency on time or missed a few days o I missed a few days while making the transition from residency to fellowship o I missed a few days while making the transition from one fellowship to another o Did any of these affect the duration of your training or start date? o Not applicable o Due to mandatory quarantine o Due to travel ban o Due to visa issue/ visa office closing o Other…
8.	How did you prepare yourself for the assigned clinical tasks? o Not applicable o Hospital provided a workshop o Team up with resident of a clinical specialty o I had an internship in a clinical specialty o I had a residency training in a clinical specialty o Other…
9.	How did pandemic affect the duration of your training? o No effect o It extended my training o I couldn’t start my residency on time or missed a few days o I missed a few days while making transition from residency to fellowship o I missed few days while making transition from fellowship to another fellowship
10.	Factors affecting the duration of your training or start date? o Not applicable o Due to mandatory quarantine o Due to travel ban o Due to visa issue/ visa office closing o Yes, because of a mandatory hospital policy Other…
11.	Did you take a COVID-19 antigen or antibody test? o No o Yes, because of self-concern o Yes, because I participated in study o Yes, because the hospital was offering it free of cost o Yes, because of a mandatory hospital policy
12.	What was the result of your COVID-19 test? o Not applicable o My test was negative o I tested positive for antigen test o I tested positive for antibody test o I wish to keep it confidential
13.	Did you experience any change in sign-out with your attending? o No change, we still do dual scope sign-out o We sign-out at multi-headed scope to maintain social distance o Virtual sign-out via zoom, team, webex etc. o Pass the slides to attending and get feedback over the phone o Dual-scope with mask on o Dual-scope with see through partition o All of the above o Other…
14.	Did your assigned presentation/journal club change? o I wasn’t assigned any presentation during this period o No change o Yes, it got canceled o Yes, it got rescheduled to a later date o Yes, it moved to a virtual presentation platform o Were there any changes with your training program educational lectures? o No change
15.	Were there any changes with your training program educational lectures? o No change o All lectures moved to be virtual o Cancellation of all lectures o Cancellation of a few lectures before moving to virtual o Other…
16.	Did you experience any cancellation of grand rounds by visiting guest speakers? o Not aware o Yes, cancellation of all o None o Cancellation only if speaker traveling out of city
17.	Did you experience a change in slide volume? o No change o Slight decrease in volume o Significant decrease in volume
18.	How did you make up for the lost educational opportunity due to a decrease in slide volume? o Not applicable o Reviewed study slide set o Used online virtual slides o I feel I am not able to make up for the lost educational opportunity o Other…
19.	Were your interviews affected during this pandemic? o No, I didn't have any job or fellowship interview during this period o Yes, it got canceled o Yes, it got rescheduled for later o Yes, it became a virtual or phone interview o Yes, I pulled out from interview because of travel safety concerns
20.	How did the pandemic affect you writing the American Board of Pathology certification exam? o Not applicable o I withdrew from the exam due to safety concerns o I withdrew from the exam due to a change in exam time and it interfered with my busy fellowship training o Other…
21.	How did the pandemic disrupt your future plans? o Not applicable o Not affected o I wanted to apply for a fellowship in a different city but now I will stay in the same city/program o I wanted to apply for a job but because of hiring freezes I am pursuing another fellowship o Other…
22.	How did the pandemic affect your social media activity related to pathology? o No Change o I don't have any social media account o I have an account, but I am not active o I created a social media account during the pandemic for social purposes o I created a social media account during the pandemic to use it a tool for pathology o I am very active on social media and frequently post topics related to pathology o I follow pathology-related topics on social media, but I rarely post by myself o I have become more active during the pandemic
23.	How often do you use online resources for training and learning purposes? o I don't use any online resources at all o I rarely use o I frequently use o I have been using it, but frequency increased during pandemic o Tremendous increase in use during pandemic
24.	Which online resource have you used the most? o CAP virtual lecture o USCAP courses offered free to residents o Pathcast o Pathpresenter o Virtual slide box - University of Michigan o Surgical pathology unknown conference - Johns Hopkins o Other…
25.	Any effects due to being pregnant during the pandemic? o Not applicable o Program allowed me to work remotely, at least for some time o Made changes in my rotation schedule to minimize the risk of exposure o I took time off from work o No special arrangement, I kept working as usual o Other…
26.	Any effects to taking care of children during this time, especially after the closing of daycare and schools? o Not applicable o Took help of parents/grandparents o Help from close family, friends and neighbors o Hospital made special arrangements o Got help from medical student volunteer drive o Forced to take off in order to take care of kids o Allowed to work from home, at least for a few days o Able to manage because my spouse/ partner works from home or is a homemaker o Other…
27.	Any family separation during the pandemic? o Not applicable o Not at all o I stayed in hotel or accommodation provided by hospital, at least for a few days o I did not visit my family living in another city due to safety concerns o I did not visit my family living in another city due to travel guideline by the hospital o I did not visit my family living in another city due to travel restrictions by government o Other…
28.	Any changes in rotation/ schedule due to the pandemic? o No change at all o Minimal changes o Some extra coverage due to co-trainee's absence due to COVID-19 factors o Major reshuffle or changes
29.	Please share any unique situation, incidence, innovative idea, or approach you experienced related to your pathology training that you faced during this pandemic.
